# How to design a dose-finding study on combined agents: Choice of design and development of R functions

**DOI:** 10.1371/journal.pone.0224940

**Published:** 2019-11-11

**Authors:** Monia Ezzalfani

**Affiliations:** Institut Curie, PSL Research University, Biometry Unit, Paris, France; University of Pittsburgh Graduate School of Public Health, UNITED STATES

## Abstract

**Background:**

In oncology, the aim of dose-finding phase I studies is to find the maximum tolerated dose for further studies. The use of combinations of two or more agents is increasing. Several dose-finding designs have been proposed for this situation. Numerous publications have however pointed out the complexity of evaluating therapies in combination due to difficulties in choosing between different designs for an actual trial, as well as complications related to their implementation and application in practice.

**Methods:**

In this work, we propose R functions for Wang and Ivanova’s approach. These functions compute the dose for the next patients enrolled and provide a simulation study in order to calibrate the design before it is applied and to assess the performance of the design in different scenarios of dose-toxicity relationships. This choice of the method was supported by a simulation study which the aim was to compare two designs in the context of an actual phase I trial: i) in 2005, Wang and Ivanova developed an empirical three-parameter model-based method in Bayesian inference, ii) in 2008, Yuan and Yin proposed a simple, adaptive two-dimensional dose-finding design. In particular, they converted the two-dimensional dose-finding trial to a series of one-dimensional dose-finding sub-trials by setting the dose of one drug at a fixed level. The performance assessment of Wang’s design was then compared with those of designs presented in the paper by Hirakawa et al. (2015) in their simulation context.

**Results and conclusion:**

It is recommended to assess the performances of the designs in the context of the clinical trial before beginning the trial. The two-dimensional dose-finding design proposed by Wang and Ivanova is a comprehensive approach that yields good performances. The two R functions that we propose can facilitate the use of this design in practice.

## Introduction

In oncology, the aim of dose-finding phase I studies is to find the maximum tolerated dose (MTD) for further studies. Currently, most designs use the number of dose-limiting toxicities (DLTs) as a primary endpoint to estimate the MTD. The MTD is then defined as the dose associated with an acceptable estimated probability of DLTs. Combining two or more agents to improve antitumoral activity and therefore efficacy is common. However, Phase I trials on such combinations, aiming to find the MTD for the combination (also rated by MTD, in order to simplify the rating) are more difficult and complex than those on conventional single agents. In single-agent trials, the MTD is based on the paradigm of an increasing dose-toxicity relationship. The continual reassessment method (CRM) is widely used in this case [1]. However, in studies on combinations, the ordering of toxicity probabilities between all combinations is not known prior to the study. For instance, the combination of two agents with three and four dose levels respectively, leads to 12 dose combinations. The ordering is only partially known among all these combinations. This leads to uncertainty in the ordering of monotonic toxicities for some dose combinations. This paper was prompted by the phase I VINILO trial, an international phase I trial evaluating the combination of vinblastine and nilotinib in children and adolescents with refractory or recurrent low-grade glioma (ClinicalTrials.gov Identifier: NCT01884922). It was planned to explore respectively four and three dose levels for vinblastine (from 3 to 6 mg/m^2^) and nilotinib (115 to 350 mg/m^2^). The study received regulatory approval in January 2013 and officially started in France in May 2013. The protocol was amended in 2014. The main modification concerned the dose-escalation method to apply the method published by Wang and Ivanova [2], whereas the initial version of the protocol used the method published by Yuan and Yin [3]. Both designs correspond to the particularities of the VINILO trial, which are detailed in the next section, and the choice of the final design was based on the simulation study, which will be presented here.

Indeed, for studies testing combinations, many designs have been proposed, either algorithm-based or model-based [2, 4–7]. For example, Wages et al. proposed a partial order CRM design [7], which is able to identify the MTD based on various possible orderings with a partial order between combinations. This ordering between combinations is assumed to be monotonic and increases with the dose. Ivanova and Wang proposed an ‘up-and-down algorithm’ method based on isotonic regression to estimate a set of possible MTDs [4]. Mandrekar et al. developed a model-based design considering both the toxicity and efficacy of each agent to identify the optimal dose [5, 6]. Wang and Ivanova developed a three-parameter model-based method for which the parameters were estimated using Bayesian inference [2]. This list from the literature is not exhaustive and many other methods exist. Some authors have compared different methods in simulation studies. Riviere et al. [8] compared two algorithm-based [4, 9] aand four model-based dose-finding methods [2, 7, 10] in a simulation using 10 realistic scenarios describing different dose-toxicity relationships. One of their conclusions was that the model-based methods performed better than the algorithm-based methods. This point corresponds to the findings in single-agent studies [11]. Hirakawa et al. proposed a comparative study on phaseI combination therapies [12]; they focused only on model-based designs in which the primary interest was to find the MTD for only one combination. One of their conclusions was that the average performance of methods across the 16 scenarios used in their simulation study varied depending on (i) whether the dose-combination matrix is square or not; (ii) whether the true MTDs exist within the same group along the diagonals of the dose-combination matrix; and (iii) the number of true MTDs.

In the setting we encountered in the VINILO trial, we propose an approach to designing a dose-finding study on a combination of agents. Our presentation of this work is limited to the two designs used in VINILO [2, 3]: i)Wang and Ivanova developed an empirical three-parameter model-based method for which the parameters were estimated using Bayesian inference. The dose assigned for the next patients was defined based on the neighboring doses considering a two-dimensional approach, ii) Yuan and Yin proposed a simple, adaptive two-dimensional dose-finding design that can accommodate any type of single-agent dose-finding method. In particular, they converted the two-dimensional dose-finding trial to a series of one-dimensional dose-finding sub-trials by setting the dose of one drug at a fixed level. Sub-trials were then conducted sequentially using the CRM. The performances of these two approaches was compared under different scenarios in a simulation study in the context of the VINILO trial. We then compared the performance of Wang’s design with those presented in the paper by Hirakawa et al. [12] in the context of their simulation studies. Six methods were used: the method based on a copula-type model, termed the YYC [10], the method using a hierarchical Bayesian model, termed the BW method [13], the likelihood-based dose-finding method using a shrinkage logistic model, termed the HHM method [14], the likelihood-based CRM for partial ordering, termed the WCO method [15], the order-restricted inference method of Conaway et al., termed CDP [16], and the Bayesian dose-finding design based on the logistic model termed RYDZ, [17].

While several other methods exist, they are rarely used in practice [8], mainly due to complications related to their implementation and the lack of simple tools. We propose a tool involving two R functions, for use with the Wang and Ivanova design: the two-dimensional dose-finding in discrete dose space [2]. The choice was prompted by the fact that: i) among the designs compared by Riviere et al., Wang’s method performed well in terms of identifying the MTD, and ii) our simulation studies yielded good performances with this method.

The Methods are presented in Section 2. In Section 3, we present the comparison study in the context of the VINILO trial. We report the results in Section 4. In Section 5, two R functions are described, one to run the simulation study in order to assess the performance of the method in various scenarios, and one to compute the dose for the next patients in a phase I trial. In Section 6, the conclusions and discussion are presented.

## Materials and methods

### Motivation for the research

**Fig 1:**
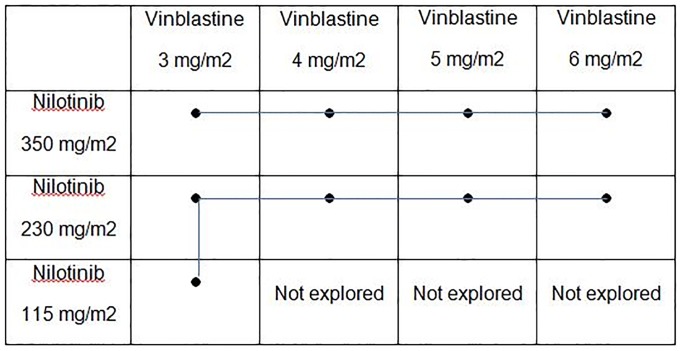
Combination of vinblastine and nilotinib explored in the phase I dose-escalation trial, VINILO.

The VINILO trial is an international phase I trial evaluating the combination of vinblastine and nilotinib in children, adolescents and young adults with low-grade gliomas in progression after first-line treatment with chemotherapy or radiotherapy. The aim of the trial is to determine the MTDs of nilotinib and vinblastine when used in combination for phase II trials, i.e. the dose associated with an estimated probability of DLT close to 20%. It was planned to explore four and three dose levels respectively for vinblastine (from 3 to 6 mg/m^2^) and nilotinib (115 to 350 mg/m^2^). A total of 12 dose levels could potentially be explored, however, after discussion with the principal investigator, the dose levels of 4, 5 and 6 mg for vinblastine combined with the dose level of 115 mg for nilotinib were not explored (see Fig 1). The trial started in France in 2013 using Yuan’s approach, which converts the two-dimensional dose-finding trial into a series of one-dimensional dose-finding sub-trials by setting the dose of one drug at a fixed level. The protocol was amended in 2014. The main change concerned the dose-escalation method to follow the method published by Wang and Ivanova [2], whereas the initial version of the protocol had proposed the method published by Yuan and Yin in 2008 [3]. The decision was based on the simulation study, which we will present here.

### Two-dimensional dose finding in discrete dose space

Wang and Ivanova proposed an approach based on an empirical model using a two-dimensional dose finding design in a Bayesian framework.

Let us consider two agents to be evaluated: Agent A where a set {*s*_1_, …*s*_*J*_} of doses will be evaluated, and Agent B where a set {*t*_1_, …*t*_*K*_} of doses will be evaluated, with *K* < *J*. The goal of Wang’s approach is to find a collection of dose combinations that yield the target probability of toxicity, for each dose level of Agent B *t*_*k*_.

Wang and Ivanova proposed a dose-combination model with interaction term as:

$$\begin{array}{c} \Psi \left(s_{j},t_{k},\theta \right)=\Pi_{jk}=1-{\left(1-a_{j}\right)}^{\alpha }{\left(1-b_{k}\right)}^{\left(\beta +\gamma *log\left(1-a_{j}\right)\right)}. \end{array}$$

With *θ* = (*α*, *β*, *γ*): the vector of parameters to be estimated, where *γ* describes the interaction between the two agents.

To satisfy the assumption of monotonicity of toxicity, the parameters should satisfy the following constraints: *α* > 0, *β* > 0, and *γ* < 0.

The vectors *a*_*j*_ and *b*_*k*_ were used in place of actual doses, with 0 < *a*_1_ < … < *a*_*J*_ < 1 and 0 < *b*_1_ < … < *b*_*K*_ < 1. These vectors were considered as initial guesses which corresponded to the “desirable range of toxicity probabilities for testing” [1].

The authors proposed to estimate the parameters of the model using a Bayesian framework. The parameter *θ* is assumed to follow a joint prior distribution with density *g*(*θ*). The posterior distribution of *θ* given toxicity data from the first *i* subjects is:

$$\begin{array}{c} f\left(\theta |D_{i}\right)=\frac{f\left(D_{i}|\theta \right)g\left(\theta \right)}{\int f\left(D_{i}|\theta \right)g\left(\theta \right)d\theta } \end{array}$$

where *f*(*D*_*i*_|*θ*) is the joint conditional density of the observed responses given *θ*:

$$\begin{array}{c} f\left(D_{i}|\theta \right)=\prod_{l=1}^{i}\Psi {\left(s_{j},t_{k},\theta \right)}^{y_{l}}{\left(1-\Psi \left(s_{j},t_{k},\theta \right)\right)}^{\left(1-y_{l}\right)} \end{array}$$

After the inclusion of each new cohort, the posterior mean of the probability of toxicity was to be updated and the authors recommended making the next dose assignment to a combination adjacent to the current one. If the current dose was (j, k), the next dose assigned was a combination from the set::

{(*j* − 1, *k*), (*j* + 1, *k*), (*j*, *k*), (*j*, *k* + 1), (*j*, *k* − 1), (*j* + 1, *k* − 1), (*j* − 1, *k* + 1)}; 1 ≤ *j* ≤ *J*, 1 ≤ *k* ≤ *K*.

Due to ethical constraints, combinations where both agents were increased at the same time (*j* + 1, *k* + 1) were not permitted.

At the end of the trial, the MTD was defined, for each dose level of Agent B, as the dose combination where the estimated probability of toxicity was closest to the target.

Wang and Ivanova proposed a start-up rule in order to gather information before estimating parameters. Patients in phase I studies are allocated to doses sequentially. In practice, the first group of the start-up is treated at the lowest dose combination (*s*_1_, *t*_1_). The start-up phase is then conducted according the following rules: i) if no toxicity is observed, the dose of the first agent is escalated; ii) if at least one toxicity is observed, the start-up is terminated at the current level (i,j), and the next dose combination is (i—2, j + 1). The start-up is terminated if all dose levels of Agent B were explored. At the end of the start-up, the vector of initial guesses is used to obtain the estimates of the toxicity rate for each combination. For safety’s sake in the dose-escalation process, we proposed to start the two-dimensional design at the lowest level of Agent B at a dose combination with the probability of toxicity closest to the target toxicity.

We note that the start-up rule can be defined depending on the context of the clinical trial, for example priority can be given to exploring certain dose combinations. The start-up rules should be defined before beginning the trial.

### Sequential CRM for two-dimensional dose-finding designs

Yuan and Yin proposed to convert the dose-escalation design for dual agents into k one-dimensional dose-escalation trials, with k being the number of dose levels for the second agent, which had fewer dose levels to be explored. Each one-dimensional trial is referred to as a sub-trial, where the dose of the second drug is set at a fixed level, and the dose of the first drug varies. In this design, the idea is to first divide the entire two-dimensional trial into groups of sub-trials and then conduct the sub-trials sequentially in a specific order. The intermediate-dose sub-trial is run first, in order to find the MTD. The MTD that has been determined in the completed sub-trial can be used as the truncation boundary to shrink the search space of other sub-trials that have not yet been carried out. The low-dose and high-dose sub-trials were then run simultaneously. For example, if dose level 3 is the MTD in the intermediate sub-trial, then based on the assumption of monotonicity, the MTD must be higher than or equal to dose level 3 in the lower sub-trial, and the MTD must be lower than or equal to dose level 3 in the higher trial. In other words, determining dose level 3 as the MTD in the intermediate sub-trial shrinks the number of patients in the lower and higher sub-trials, by eliminating “inadmissible” doses.

The two sub-trials are not independent of the main trial because the vectors of initial guesses of the low-dose and high-dose sub-trials are proportional to the posterior toxicity probability of the main sub-trial. For each sub-trial, the conventional Bayesian CRM with a one-parameter empirical model is used [1]. At the end of the trial, this method provides an MTD for each dose level of the second agent. The MTD is defined as the dose combination with an estimated probability of toxicity closest to the target.

## Simulation study

### Design parameters

The performances of Wang and Yuan approaches were compared under different scenarios in a simulation study in the context of the VINILO trial. After discussion with the principal investigator, the dose levels of 4, 5 and 6 mg of vinblastine combined with the dose level of 115 mg of nilotinib were not explored (see Fig 1). As the trial was started using Yuan’s approach, no start-up rule was proposed for Wang’s method. To be consistent across the different methods, the trial was to stop when the prespecified number of patients, n, was exhausted. The number of patients was set at 40, which was the capacity of trial recruitment. Using Yuan’s approach, two sub-trials were considered, and the number of patients was divided into 24 patients in the main trial and 16 in the high-dose sub-trial. The cohort size was 3 patients. The target probability of DLTs was set at 20%.

For each scenario, we simulated 1000 repetitions of a trial. Skipping of dose levels during dose escalation was not permitted. No intra-patient dose escalation was permitted. At least 2 patients fully observed with no DLTs were required at a given dose before dose escalation. We used the same definition of the MTD in both methods, i.e. the dose associated with an estimated DLT probability closest to, but below, the target DLT. The target was set at 20%.

For the Wang and Ivanova approach, the parameter *γ* was set at 0 assuming no interaction between the two agents nilotinib and vinblastine. The prior distribution for the parameters (*α*, *β*) was the product of two independent Exponential with a mean of 1. The Bayesian estimation was based on 5000 Monte Carlo iterations with no start-up.

For the Yuan and Yin approach, as recommended by the authors, a non-informative prior distribution Gaussian (0, 2) was assigned in the Bayesian computation, and the proportionality coefficients for the initial guesses in the sub-trial were set at (0.85, 1.15), as recommended by the authors. We used the dfcrm R package to apply the CRM for each sub-trial.

The initial guesses, as proposed by Wang, were set at (0.05, 0.1, 0.2, 0.3) for Agent A, and (0.05, 0.1, 0.2) for Agent B.

As the initial comparison concerned the methods of both Wang and Yuan in the VINILO context, we completed the performance assessment of Wang’s design, which was used in VINILO trial, by comparing it with those of designs presented in the paper by Hirakawa et al. [12] in the context of their simulation studies. Please refer to their paper for the designs compared and for the 16 scenarios studied in their simulation studies. We used the simulation setting of Hirakawa et al. [12]; the target toxicity was set to 0.3, no stopping rule was specified, and the pre-specified maximum sample size was set at 30. Each simulation study consisted of 1000 trials. The values of the prior toxicity probabilities for Agent A and Agent B were set at the same level as the prior toxicity probabilities for both agents as used in their paper (i.e. (0.15, 0.3) for J = 2, (0.1, 0.2, 0.3) for J = 3, (0.075, 0.15, 0.225, 0.3) for J = 4, and (0.06, 0.12, 0.18, 0.24, 0.3) for J = 5).

### Sensitivity analysis

A sensitivity analysis was performed to evaluate the performance of the method where: i) the proportionality coefficients were set at (0.85,1.5) and (0.85,2) instead of (0.85,1.15), and ii) different initial guesses using the R function “getprior” in the dfcrm package were used. As stated by Yin G and Lin R [18], typically, the maximum dose for each drug in a combination trial cannot exceed their individual MTDs, and as a result, the remaining lower doses can only be fractions of the corresponding MTDs. Thus, the maximum toxicity probability for each drug in the combination typically cannot exceed 20%, as synergism in DLTs is often a major concern.

### Parameters of the scenarios

The simulation study was performed using the scenarios reported by Wang and Yuan. A total of 8 scenarios were investigated (Table 1). The first four were based on those reported by Wang and the other four were based on those reported by Yuan, with a target probability of DLT set at 20% in all scenarios. These scenarios spanned a broad range of hypotheses for dose-toxicity relationships in the context of VINILO.

**Table 1:** Toxicity scenarios for the two-agent trial with target probability of toxicity 0.2. MTDs are in bold.

	*s*_1_	*s*_2_	*s*_3_	*s*_4_	*s*_5_	*s*_6_
**Scenarios in Wang’s paper**						
**Scenario 1**						
Level 3	0.08	0.13	0.20	0.29		
Level 2	0.05	0.08	0.13	0.20		
Level 1	0.03					
**Scenario 2**						
Level 3	0.05	0.08	0.11	0.15		
Level 2	0.04	0.06	0.09	0.13		
Level 1	0.04					
**Scenario 3**						
Level 3	0.20	0.30	0.41	0.53		
Level 2	0.10	0.20	0.25	0.32		
Level 1	0.03					
**Scenario 4**						
Level 3	0.20	0.40	0.47	0.56		
Level 2	0.08	0.13	0.20	0.32		
Level 1	0.03					
**Scenarios in Yuan’s paper**						
**Scenario 5**						
Level 3	0.20	0.30	0.40	0.50		
Level 2	0.10	0.20	0.30	0.40		
Level 1	0.05					
**Scenario 6**						
Level 3	0.10	0.20	0.30	0.40		
Level 2	0.05	0.12	0.20	0.30		
Level 1	0.04					
**Scenario 7**						
Level 3	0.30	0.42	0.52	0.62		
Level 2	0.20	0.30	0.40	0.50		
Level 1	0.02					
**Scenario 8**						
Level 3	0.11	0.21	0.31	0.42		
Level 2	0.10	0.20	0.30	0.40		
Level 1	0.08					

### Simulation of trials and metrics of comparison

In the context of VINILO, as the aim of the trial is to identify one MTD at the end of the trial, the methods were compared in terms of the percentage of dose recommendations, in particular the percentage of correct selection (PCS). The average number of observed DLTs at each dose level and the average number of patients treated at each dose level were also calculated (results available on request).

## Results

The distribution of the recommended dose is presented in Table 2. Wang’s method yielded a good performance with a PCS varying from 45% to 72%. In 6 of the 8 scenarios studied, Wang’s method presented a PCS higher than that with Yuan’s approach, with a difference in PCS varying from 7.6 to 44.6%. The control of overdosing was better with Wang’s method in 6 of the 7 cases concerned.

**Table 2:** Distribution of the maximum tolerated dose. Percentages at the maximum-tolerated dose combinations (or combinations closest to them) are in bold.

	Wang’s method (with no start-up)				Yuan’s method			
	*s*_1_	*s*_2_	*s*_3_	*s*_4_	*s*_1_	*s*_2_	*s*_3_	*s*_4_
**Wang’s scenarios**								
**Scenario 1**								
Level 3	7.8	12.4	29.8	19.5	6.3	15.9	16.2	12.5
Level 2	0.0	1.8	11.0	17.7	0.7	6.5	18.1	23.7
Level 1	0.0				0.0			
**Scenario 2**								
Level 3	1.8	2.6	12.8	71.9	2.1	7.7	13.3	27.3
Level 2	0.0	0.2	1.7	9.0	0.8	4.4	12.3	32
Level 1	0.0				0.0			
**Scenario 3**								
Level 3	17.7	4.3	3.2	0	18.5	9.4	1.7	0
Level 2	10.4	39.9	22.3	2	11.3	27.9	21.3	9.9
Level 1	0.2				0.0			
**Scenario 4**								
Level 3	22.9	3.5	1.8	0.1	21.2	7.1	0.7	0.2
Level 2	2.4	25.3	40.5	3.5	3.8	20.7	31.3	15.0
Level 1	0.0				0.0			
**Yuan’s scenarios**								
**Scenario 5**								
Level 3	16.6	4.5	2.5	0.0	17.8	9.4	2.4	0.0
Level 2	13.0	46.2	16.2	0.7	13.1	33.3	19.7	4.3
Level 1	0.3				0.0			
**Scenario 6**								
Level 3	22.1	14.2	16.0	2.3	10.9	17	8.3	1.1
Level 2	1.3	12.6	26.7	4.8	2	18.3	28.7	13.6
Level 1	0.0				0.0			
**Scenario 7**								
Level 3	4.3	0.2	0.0	0.0	14.7	3.7	0.0	0.0
Level 2	53.2	31.6	2.2	0.1	39.9	33.6	6.8	1.3
Level 1	8.4				0.0			
**Scenario 8**								
Level 3	15.6	6.4	6.3	0.2	11.7	17.7	3.4	0.4
Level 2	11.4	41.8	16.7	1.2	15.2	30.6	17.6	3.4
Level 1	0.4				0.0			

The percentage of patients treated at each dose level was compared. The number of patients treated at the MTD per dose level of the second dose was better with Wang’s method (results available on request).

The performances of Wang’s method compared to the designs used in the paper by Hirakawa for the 16 scenarios studied are presented in Table 3 [12, 19]. The results are presented in terms of PCS and the average number of patients allocated to the MTD. The operating characteristics of Wang’s method were inferior to those of the designs presented in the paper by Hirakawa, with an average difference of -11% in the PCS. Over-dosing was well controlled in Wang’s method. The average number of patients allocated to the MTD was excellent with Wang’s method, with a difference of +19. Wang’s method appeared to be more conservative in the dose escalation process, with many more patients allocated to the MTD-1, in particular when it was the next closest dose to the target, leading to a conservative method, which may have been due to the start-up step used at the beginning of the trial. In fact, using the start-up steps, doses starting at the lowest dose level can be explored, and then the first agent can be explored while keeping the dose of the second agent at a fixed level until the first DLT occurs. This rule can however consume a large number of patients at sub-therapeutic doses before exploring the MTD, and can thus lead to a shortage of patients to explore the MTD. For example, in scenarios 5 to 7 where the first doses had very low toxicity, many patients were used during the start-up before starting the two-dimensional trial. In these scenarios, a total of 16 doses were explored for 30 patients, resulting in an average of two patients per dose.

**Table 3:** Summary of the operating characteristics of the six methods in all scenarios in paper by Hirakawa. For reminder: YYC, Yin and Yuan [10]; CDP, Conaway, Dunbar, and Peddada [16]; BW, Braun and Wang [13]; WCO, Wages, Conaway, and O’Quigley [15]; HHM, Hirakawa, Hamada, and Matsui [14]; RYDZ, Riviere et al. [17]; Wang’s method, Wang, K. and Ivanova, A. [2]; Wang’s variant, Wang’s method without start-up step.

	Scenarios																
Method	1	2	3	4	5	6	7	8	9	10	11	12	13	14	15	16	Average
	Recommendation rates for true MTD dose combinations (%)																
YYC	44	70	26	6	59	44	33	5	39	49	72	2	30	18	37	2	34
CDP	62	81	25	29	80	43	46	23	53	41	92	38	51	39	34	22	47
BW	50	66	24	36	58	37	28	24	39	38	83	29	38	33	33	20	40
WCO	56	73	32	31	77	40	37	23	49	48	86	36	39	44	41	26	46
HHM	45	66	28	38	64	49	31	29	47	46	70	22	30	44	42	20	42
RYDZ	48	85	30	50	82	50	33	42	35	35	84	40	34	57	35	24	48
Wang’s method	50	70	28	20	53	33	25	15	36	44	79	30	25	34	31	15	37
Wang’s variant	51	73	30	26	54	38	31	53	35	49	78	30	26	40	37	15	42
	Average number of patients allocated to true MTD combinations																
YYC	8	14	6	1	12	9	5	1	6	10	10	1	6	4	8	0	6
CDP	14	17	5	6	17	9	11	5	12	10	24	8	11	8	8	5	11
BW	11	14	5	9	12	8	6	6	10	8	23	5	8	8	6	4	9
WCO	14	16	7	7	16	10	7	5	11	12	21	8	8	10	9	5	10
HHM	11	14	6	6	9	8	3	5	8	9	15	5	5	7	7	3	8
RYDZ	11	15	4	10	14	10	5	8	8	7	20	7	6	12	5	3	9
Wang’s method	38	60	18	16	41	30	24	14	32	27	67	27	24	29	29	11	30
Wang’s variant	45	60	20	22	53	39	32	16	34	23	78	39	37	47	22	9	36

We ran the same simulations using a variant of Wang’s method with no start-up step. The results are presented in Table 3, noted as “Wang variant”. The operating characteristics of the Wang variant were similar to those of the designs presented in the paper by Hirakawa, with an average difference in PCS less than 8%, with the best PCS in scenarios 8 and 10. The average number of patients allocated to the MTD was excellent with Wang’s method, with a difference of +25 patients treated at the MTD. We ran the same simulations using 50 patients instead of 30, and the results were improved by more than 10% in all of the scenarios (results available on request).

### Sensitivity analysis

We studied the operating characteristics of Yuan’s method with different values for the proportionality coefficients for the initial guesses in the sub-trials. We considered pairs of values (0.85, 1.5) and (0.85, 2) instead of (0.85, 1.15). Similar results were obtained (results available upon request). We also compared the performance of Wang’s method with different initial guesses. The results were similar in most scenarios (results available upon request).

## Program description

We proposed scripts using Wang’s method. We presented two functions: i) Wang’s step-by-step (SBS) design, which computes the dose for the next patient in an actual phase I trial, and ii) Wang’s simulation (SIM) design, which provides simulation replicates of a phase I trial using the Wang and Ivanova design. Both functions were written in R language. No downloading of R packages was required. The functions must be loaded in each work session and require several arguments, detailed below.

### R function for phase I trials: SBS_design

The function SBS_design is used to compute a dose for the next patient in a phase I trial based on the two-dimensional dose-finding design developed by Wang and Ivanova. The R function developed can be used with or without start-up rules.

**Arguments**

SBS_design(cur_d1,cur_d2,nsamp, nd1, nd2, prior_1, prior_2, target, npts, ntox, ntot)

cur_d1: Dose level of Agent A to be assigned for the next patients

cur_d2: Dose level of Agent B to be assigned for the next patients

nsamp: The number of iterations needed for the MCMC

nd1: The number of dose levels of Agent A

nd2: The number of dose levels of Agent B

prior_1: A vector of initial guesses of toxicity probabilities associated with the doses for Agent A

prior_2: A vector of initial guesses of toxicity probabilities associated with the doses for Agent B

target: The target DLT rate

npts: A matrix of the number of patients treated at each dose level

ntox: A matrix of patient outcomes; number of DLTs observed at each dose level. ≥ 1 indicates one or more toxicities, 0 otherwise.

ntot: The number of patients enrolled

**Value**

cur_d1: Dose level of Agent A to be assigned for next patients

cur_d2: Dose level of Agent B to be assigned for next patients

npts: A matrix of the number of patients treated at each dose level

ntox: A matrix of patient outcomes; number of DLTs observed at each dose level. ≥ 1 indicates one or more toxicities, 0 otherwise.

MTD_lv: Updated estimate of the MTD for each dose level of Agent B.

**R code**

SBS_design = function(cur_d1, cur_d2, nsamp, nd1, nd2, prior_1, prior_2, target, npts, ntox, ntot)

{

print(“*****************************************************************”)

print(“*    R function for two-Dimensional Dose Finding           *”)

print(“*    in Discrete Dose Space of Wang K and Ivanova A        *”)

print(“*       (M. Ezzalfani* & T. Filleron)             *”)

print(“*****************************************************************”)

print(“* *Institut Curie                         *”)

print(“* 25 rue d’Ulm                           *”)

print(“* 75005                              *”)

print(“* France                             *”)

print(“*****************************************************************”)

print(“ ”,quote = F)

# Data Check

print(“+--------------------+”)

print(“|    Data Check    |”)

print(“+--------------------+”)

quote(“ ”)

  # For nd1 and nd2

## nd1 et nd2 are numeric

if(is.numeric(nd1)==FALSE) stop(“nd1 must be numeric”)

if(is.numeric(nd2)==FALSE) stop(“nd2 must be numeric”)

## nd1 nd2 entier>0

if(ceiling(nd1)-nd1!=0 | nd1<=0) stop(“nd1 must be Integer (>0)”)

if(ceiling(nd2)-nd2!=0 | nd2<=0) stop(“nd2 must be Integer (>0)”)

## nd1 >= nd2

if(nd1 < nd2) stop(“nd1 must be superior to nd2”)

## nsamp entier>0

if(is.numeric(nsamp)==FALSE) stop(“nsamp must be numeric”)

if(ceiling(nsamp)-nsamp!=0 | nsamp<=0) stop(“nsamp must be Integer (>0)”)

## ntot

if(is.numeric(nsamp)==FALSE) stop(“ntot must be numeric”)

if(ceiling(ntot)-ntot!=0 | ntot<=0) stop(“ntot must be Integer (>0)”)

# For prior_1 prior_2

## For prior_1

if(is.vector(prior_1)==FALSE)stop(“prior_1 must be a vector (Numeric Argument)”)

if(length(prior_1)!=nd1)stop(“prior_1 must be a vector (lengh nd1)”)

if(min(prior_1)<0 | max(prior_1)>1) stop(“Verify probability prior_1 (<0 or >1)”)

## For prior_2

if(is.vector(prior_2)==FALSE)stop(“prior_2 must be a vector (Numeric Argument)”)

if(length(prior_2)!=nd2)stop(“prior_2 must be a vector (lengh nd2)”)

if(min(prior_2)<0 | max(prior_2)>1) stop(“Verify probability prior_2 (<0 or >1)”)

# For cur_d1 cur_d2

## For cur_d1

if(is.numeric(cur_d1)==FALSE)stop(“cur_d1 must be a numeric argument (Numeric Argument)”)

if(cur_d1<0 | cur_d1>nd1) stop(“Verify cur_d1)”)

## For cur_d2

if(is.numeric(cur_d2)==FALSE)stop(“cur_d2 must be a numeric argument (Numeric Argument)”)

if(cur_d2<0 | cur_d2>nd2) stop(“Verify cur_d2)”)

# ntox npts

## npts

if (is.matrix(npts)==FALSE)stop(“npts need to be matrix (dimension nd2xnd1)”)

if (dim(npts)[1]!=nd2 | dim(npts)[2]!=nd1)stop(“Verify dimensions matrix npts (dimension nd2xnd1)”)

## ntox

if (is.matrix(ntox)==FALSE)stop(“ntox need to be matrix (dimension nd2xnd1)”)

if (dim(ntox)[1]!=nd2 | dim(ntox)[2]!=nd1)stop(“Verify dimensions matrix ntox (dimension nd2xnd1)”)

## ntox<=npts

temp <- npts-ntox

if (sum(temp<0)!=0)stop(“Verify matrix ntox and npts (Nb of toxicty greated than nb of patients)”)

  # Compute the probabilities pijk

 pijk_model = function(ai,bj,alpha,Beta,Gamma)

 {

  pijk <- (1—((1-ai)^alpha*(1-bj)^(Beta+Gamma*(1-ai))))

  return(pijk)

 }

 #likelihood is the joint conditional density of the observed responses (=f(D_k|theta) in the Ivanova’s paper)

 likelihood = function(alpha,Beta, Gamma,nd1,nd2,prior_1,prior_2,npts,ntox)

 {

  rst <- 1

  for(i in 1:nd1){

   for(j in 1:nd2){

    pijk <- pijk_model(prior_1[i],prior_2[j],alpha,Beta,Gamma)

    rst  <- rst * pijk^ntox[nd2-j+1,i] * (1-pijk)^(npts[nd2-j+1,i]-ntox[nd2-j+1,i])

   }

  }

  return(rst)

 }

  #Estimation_pijk: to estimate the pijk

   Estimation_pijk = function(nsamp, nd1, nd2, prior_1, prior_2, npts, ntox)

  {

   #set.seed(55)

   alpha_samp <- rexp(nsamp,1)

   Beta_samp <- rexp(nsamp,1)

   Gamma_samp <- rexp(nsamp,1)

   pijk    <- matrix(0,nrow = nd2,ncol = nd1)

   num     <- numeric(nsamp)

   f_lik    <- likelihood(alpha_samp,Beta_samp,Gamma_samp,nd1,nd2,prior_1,prior_2,npts,ntox)

   for(j in 1:nd1)

   {

    for(k in 1:nd2)

    {

     pijk_samp = pijk_model(prior_1[j],prior_2[k],alpha_samp,Beta_samp,Gamma_samp)

     pijk[nd2-k+1, j] <- sum(pijk_samp*f_lik)/sum(f_lik)

    }

   }

   return(pijk)

  }

  # Recommended_dose: is the decision rule to recommend the next dose: the dose associated with a probability the closest to the target

  Recommended_dose = function(cur_d1,cur_d2,pijk,target,npts,ntot,nd2)

  {

   next_d1 <- NA

   next_d2 <- NA

   # Next assignement is to one of the combinations from: (i+1,j), (i,j+1), (i-1, j+1), (i-1,j), (i,j-1), (i+1,j-1), et (i,j)

   next_assig <- list(c(min(max(cur_d1-1,1),nd1), cur_d2),         #(i-1,j)

               c(min(max(cur_d1+1,1),nd1), cur_d2),            #(i+1,j)

               c(cur_d1,cur_d2),                        #(i,j)

               c(cur_d1, min(max(cur_d2+1,1),nd2)),            #(i,j+1)

               c(cur_d1, min(max(cur_d2-1,1),nd2)),            #(i,j-1)

               c(min(max(cur_d1+1,1),nd1), min(max(cur_d2-1,1),nd2)), #(i+1,j-1)

               c(min(max(cur_d1-1,1),nd1), min(max(cur_d2+1,1),nd2))) #(i-1, j+1)

   next_assig      <- next_assig[!duplicated(next_assig)]

   length_next_assig   <- length(next_assig)

   difference      <- c()

   for(ll in 1:length_next_assig)

   {

    difference  <- c(difference, abs(pijk[nd2-next_assig[[ll]][2]+1, next_assig[[ll]][1]]—target))

   }

   ind_min_dist <- which.min(difference)

   next_d1    <- next_assig[[ind_min_dist]][1]

   next_d2    <- next_assig[[ind_min_dist]][2]

   return(list(next_d1,next_d2))

  }

  colnames(npts) <-paste(“dose_level”, 1:nd1,sep=“”)

  rownames(npts) <-paste(“dose_level”, nd2:1,sep=“”)

  colnames(ntox) <-paste(“dose_level”, 1:nd1,sep=“”)

  rownames(ntox) <-paste(“dose_level”, nd2:1,sep=“”)

pijk          <- Estimation_pijk(nsamp, nd1, nd2, prior_1, prior_2, npts, ntox)

if(sum(npts)<ntot)

   {

cur_d1         <- Recommended_dose(cur_d1,cur_d2,pijk,target,npts,ntot,nd2)[[1]]

cur_d2         <- Recommended_dose(cur_d1,cur_d2,pijk,target,npts,ntot,nd2)[[2]]

MTD_lv         <- NA

   }

if(sum(npts)==ntot)

   {

difference       <- abs(pijk-target)

MTD_comb      <- which(difference==min(difference,na.rm = T), arr.ind = T)

cur_d1         <- MTD_comb[2]

cur_d2         <- MTD_comb[1]

MTD_lv       <- apply(difference,1,which.min)

   }

print(“- Current Dose agent A (cur_d1):”)

print(cur_d1)

print(“- Current Dose agent B (cur_d2):”)

print(cur_d2)

print(“- Number of patients treated at each dose level (npts):”)

print(npts)

print(“- Number of DLT observed at each dose level (ntox):”)

print(ntox)

print(“- Updated estimate of the MTD for each dose level of the agent B (MTD_lv):”)

print(MTD_lv)

return(list(cur_d1 = cur_d1,cur_d2 = cur_d2,MTD_lv = MTD_lv,pijk = pijk,npts = npts,ntox = ntox,ntot))

}

**Example**

The example concerns a phase I trial evaluating two agents, Agent A with five dose levels and Agent B with three dose levels. Thirty patients will be included in the trial, by cohorts of 3 patients. The first cohort will be allocated to dose level (1,1). Assuming that no toxicity is been observed at this dose, the next dose for the next cohort of patients is given as follows:

SBS_design(cur_d1,cur_d2,nsamp, nd1, nd2, prior_1, prior_2, target, npts, ntox, ntot)

To apply our R function, the arguments will be set as:

prior_1 ← c(0.12, 0.2, 0.3, 0.4, 0.5)

prior_2 ← c(0.2, 0.3, 0.4)

nsamp ← 1000

target ← 0.3

nd1 ← 5

nd2 ← 3

ntot ← 30

# Data

# matrix presenting the number of patients treated for each combination

npts ← matrix(0, nrow = nd2, ncol = nd1)

# matrix presenting the number of toxicities for each combination

ntox ← matrix(0, nrow = nd2, ncol = nd1)

# Cohort 1, (1,2,3)

cur_d1 ← 1 #agent A

cur_d2 ← 1 #agent B

npts[3, 1] ← 3

SBS_design(cur_d1,cur_d2,nsamp, nd1, nd2, prior_1, prior_2, target, npts, ntox, ntot)

The program first verifies consistencies in the parameters and error messages can be displayed. For example, if the length of prior_1 is superior or inferior to nd1, the program stops and signals that *“prior_1 must be a vector (length nd1)”*. If there is no problem and the parameters are considered correct, calculations can then be continued, with the following message: *“+ Parameters OK”*.

A summary of the number of patients treated at each dose level, the numbers of DLTs observed at each dose level and the next dose to be explored are then given.

### R function for simulation: SIM_design

The function SIM_design is used to generate simulation replicates of a phase I trial using the two-dimensional dose-finding design developed by Wang and Ivanova, under a specified dose-toxicity configuration.

**Arguments**

nd1: The number of dose levels of Agent A

nd2: The number of dose levels of Agent B

tox_true: A specified dose-toxicity configuration

prior_1: A vector of initial guesses of toxicity probabilities associated with the doses for Agent A

prior_2: A vector of initial guesses of toxicity probabilities associated with the doses for Agent B

target: The target DLT rate

ntot: The number of patients enrolled

ncoh_st: The number of patients enrolled in the cohort used for the starting rule

ncoh: The number of patients enrolled in the cohort used for the main design

nsamp: The number of iteration needed for the MCMC

nsim: The number of simulations

seed: Seed of the random number generator

**Value**

result_pt: Average number of patients treated at the test doses. If nsim = 1, this is a vector of length n indicating the doses assigned to the patients in the simulated trial.

result_tox: Average number of toxicities seen at the test doses. If nsim = 1, this is a vector of length n indicating the toxicity outcomes of the patients in the simulated trial.

result_MTD: Distribution of the MTD estimates. If nsim = 1, this is a single numerical value of the recommended MTD in simulated trial. If nsim = 1, this is a single numerical value of the recommended MTD in the simulated trial.

result_MTD_lv: Distribution of the MTD estimates for each dose level of Agent B. If nsim = 1, this is a single numeric value of the recommended MTD for each dose level of Agent B, in the simulated trial.

**R code**

SIM_design=function(nd1,nd2,tox_true,prior_1,prior_2,target,ntot,ncoh_st,ncoh,nsamp,Interaction,nsim,seed)

{

print(“*****************************************************************”)

print(“*     R function for simualtion two-Dimensional Dose Finding   *”)

print(“*     in Discrete Dose Space of Wang K and Ivanova A      *”)

print(“*       (M. Ezzalfani* & T. Filleron)             *”)

print(“*****************************************************************”)

print(“* *Institut Curie                         *”)

print(“* 25 rue d’Ulm                           *”)

print(“* 75005                              *”)

print(“* France                            *”)

print(“*****************************************************************”)

print(“ ”,quote = F)

# Data Check

print(“+--------------------+”)

print(“|  Data Check  |”)

print(“+--------------------+”)

quote(“ ”)

# For nd1 and nd2

## nd1 et nd2 are numeric

if(is.numeric(nd1)==FALSE) stop(“nd1 must be numeric”)

if(is.numeric(nd2)==FALSE) stop(“nd2 must be numeric”)

## nd1 nd2 entier>0

if(ceiling(nd1)-nd1!=0 | nd1<=0) stop(“nd1 must be Integer (>0)”)

if(ceiling(nd2)-nd2!=0 | nd2<=0) stop(“nd2 must be Integer (>0)”)

## nd1 >= nd2

if(nd1 < nd2) stop(“nd1 must be superior to nd2”)

# For prior_1 prior_2

## For prior_1

if(is.vector(prior_1)==FALSE)stop(“prior_1 must be a vector (Numeric Argument)”)

if(length(prior_1)!=nd1)stop(“prior_1 must be a vector (lengh nd1)”)

if(min(prior_1)<0 | max(prior_1)>1) stop(“Verify probability prior_1 (<0 or >1)”)

## For prior_2

if(is.vector(prior_2)==FALSE)stop(“prior_2 must be a vector (Numeric Argument)”)

if(length(prior_2)!=nd2)stop(“prior_2 must be a vector (lengh nd2)”)

if(min(prior_2)<0 | max(prior_2)>1) stop(“Verify probability prior_2 (<0 or >1)”)

# For tox_true

## tox_true is matrix

if(is.matrix(tox_true)==FALSE)stop(“tox_true must be a matrix (Numeric Argument)”)

## Dimension de la matrice

if(dim(tox_true)[[1]]!=nd2 | dim(tox_true)[[2]]!=nd1)stop(“tox_true must be a matrix (Dimension: nd2 x nd1)”)

## Proba of tox between 0 and 1

if(min(tox_true)<0 | max(tox_true)>1) stop(“Verify probability tox_true (<0 or >1)”)

# Target between 0 and 1

if(min(target)<0 | max(target)>1) stop(“Verify probability of tox (<0 or >1)”)

# For ntot, ncoh_st, ncoh

## ntot, ncoh_st, ncoh, nsamp are numeric

if(is.numeric(ntot)==FALSE) stop(“ntot must be numeric”)

if(is.numeric(ncoh_st)==FALSE) stop(“ncoh_st must be numeric”)

if(is.numeric(ncoh)==FALSE) stop(“ncoh must be numeric”)

if(is.numeric(nsamp)==FALSE) stop(“nsamp must be numeric”)

if(is.numeric(nsim)==FALSE) stop(“nsim must be numeric”)

## ntot, ncoh_st, ncoh entier>0

if(ceiling(ntot)-ntot!=0 | ntot<=0) stop(“ntot must be Integer (>0)”)

if(ceiling(ncoh_st)-ncoh_st!=0 | ncoh_st<=0) stop(“ncoh_st must be Integer (>0)”)

if(ceiling(ncoh)-ncoh!=0 | ncoh<=0) stop(“ncoh must be Integer (>0)”)

if(ceiling(nsim)-nsim!=0 | nsim<=0) stop(“nsim must be Integer (>0)”)

print(“+ Parameters OK”)

result_MTD    <- matrix(0, nrow = nd2, ncol = nd1)

result_tox     <- matrix(0, nrow = nd2, ncol = nd1)

result_pt      <- matrix(0, nrow = nd2, ncol = nd1)

set.seed(graine)

result_MTD    <- matrix(0, nrow = nd2, ncol = nd1)

result_MTD_lv    <- matrix(0, nrow = nd2, ncol = nd1)

result_tox     <- matrix(0, nrow = nd2, ncol = nd1)

result_pt      <- matrix(0, nrow = nd2, ncol = nd1)

Design_Wang_Ivanova = function(nd1, nd2, tox_true, prior_1, prior_2, target, ntot, ncoh_st, ncoh, nsamp, Interaction)

{

 d1_max     <- 0

 d2_max     <- 0

 ### Start-up Rule

 cur_d1    <- 1

 cur_d2    <- 1

 start_up_end <- FALSE

 ###################Other used functions############

  # gene_tox: to generate toxicity data

 gene_tox = function(ncoh,true_tox)

 {

  Z_tox = runif(ncoh)

  y_tox = as.numeric(Z_tox <= true_tox)

  return(y_tox)

 }

 #Compute the probabilities pijk

 pijk_model = function(ai,bj,alpha,Beta,Gamma)

 {

  pijk <- (1—((1-ai)^alpha*(1-bj)^(Beta+Gamma*(1-ai))))

  return(pijk)

 }

 #likelihood is the joint conditional density of the observed responses (=f(D_k|theta) in the Ivanova’s paper)

 likelihood = function(alpha,Beta, Gamma,nd1,nd2,prior_1,prior_2,npts,ntox)

 {

  rst <- 1

  for(i in 1:nd1){

   for(j in 1:nd2){

   pijk <- pijk_model(prior_1[i],prior_2[j],alpha,Beta,Gamma)

        rst  <- rst * pijk^ntox[nd2-j+1,i] * (1-pijk)^(npts[nd2-j+1,i]-ntox[nd2-j+1,i])

    }

  }

  return(rst)

 }

 #Estimation_pijk: to estimate the pijk

 Estimation_pijk = function(nsamp, nd1, nd2, prior_1, prior_2, npts, ntox,Interaction)

 {

  alpha_samp   <- rexp(nsamp,1)

  Beta_samp  <- rexp(nsamp,1)

  if (Interaction==0) {Gamma_samp <- 0}

  else {Gamma_samp <- rexp(nsamp,1)}

  pijk     <- matrix(0,nrow = nd2,ncol = nd1)

  num     <- numeric(nsamp)

  f_lik    <- likelihood(alpha_samp,Beta_samp,Gamma_samp,nd1,nd2,prior_1,prior_2,npts,ntox)

  for(j in 1:nd1)

  {

   for(k in 1:nd2)

   {

    pijk_samp = pijk_model(prior_1[j],prior_2[k],alpha_samp,Beta_samp,Gamma_samp)

    pijk[nd2-k+1, j] <- sum(pijk_samp*f_lik)/sum(f_lik)

   }

  }

  return(pijk)

 }

 ### The start-up Rule

 start_up = function(start_up_end,ntot,ncoh_st,cur_d1,cur_d2,npts,true_tox,ntox)

 {y       <- c()

 npts      <- ntox <- matrix(0, nrow = nd2, ncol = nd1)

 colnames(npts)  <-paste(“dose_level”, 1:nd1,sep=“”)

 rownames(npts) <-paste(“dose_level”, nd2:1,sep=“”)

 colnames(ntox)  <-paste(“dose_level”, 1:nd1,sep=“”)

 rownames(ntox) <-paste(“dose_level”, nd2:1,sep=“”)

 n_rem      <- ntot

 while(start_up_end == FALSE && n_rem >= ncoh_st)

 {

  d1_stup <- cur_d1

  d2_stup <- cur_d2

  y_tox <- c()

  n_rem <- n_rem-ncoh_st

  #agent 2 is in rows from 1 to nd2 beginning by the end, as in the paper

  npts[nd2-cur_d2+1, cur_d1] <- npts[(nd2-cur_d2+1), cur_d1] + ncoh_st

  true_tox <- tox_true[(nd2-cur_d2+1), cur_d1]

  y_tox  <- gene_tox(ncoh_st, true_tox)

  y    <- c(y, sum(y_tox))

  ntox[nd2-cur_d2+1, cur_d1] <- ntox[(nd2-cur_d2+1), cur_d1]+sum(y_tox)

  # if no tox observed => escalate the first agent

  if(sum(y_tox) == 0)

  {

   if(cur_d1 < nd1)

   {

    d1_stup <- cur_d1+1

   }

   if(cur_d1 == nd1 && cur_d2 < nd2)

   {

    d1_stup <- cur_d1-2

    d2_stup <- cur_d2+1

   }

   if(cur_d1 == nd1 && cur_d2 == nd2)

   {

    start_up_end <- TRUE

   }

  }

  # if at least one tox is observed

  else

  {

   if(cur_d1 > 2 && cur_d2 < nd2)

   {

    d1_stup = cur_d1-2

    d2_stup = cur_d2+1

   }

   else

   {

    start_up_end <- TRUE

   }

  }

  cur_d1 <- d1_stup

  cur_d2 <- d2_stup

 }

 ### Next dose

 pijk     <- Estimation_pijk(nsamp, nd1, nd2, prior_1, prior_2, npts, ntox,Interaction)

 dose_level_1 <- which.min(abs(pijk[nd2,1:nd1]-target))

 cur_d1 <- dose_level_1

 cur_d2 <- 1

 nused <- ntot-n_rem

 return(list(pijk = pijk,cur_d1 = cur_d1,cur_d2 = cur_d2,npts = npts,ntox = ntox,ntot = ntot,n_rem = n_rem,nused = nused,y = y))

 }

 # Recommended_dose: is the decision rule to recommend the next dose: the dose associated with a probability the closest to the target

 Recommended_dose = function(cur_d1,cur_d2,pijk,target,npts,ntot,nd2)

 {

  next_d1 <- NA

  next_d2 <- NA

  # Next assignement is to one of the combinations from: (i+1,j), (i,j+1), (i-1, j+1), (i-1,j), (i,j-1), (i+1,j-1), et (i,j)

  next_assig <- list(c(min(max(cur_d1-1,1),nd1), cur_d2),       #(i-1,j)

          c(min(max(cur_d1+1,1),nd1), cur_d2),          #(i+1,j)

          c(cur_d1,cur_d2),                    #(i,j)

          c(cur_d1, min(max(cur_d2+1,1),nd2)),          #(i,j+1)

          c(cur_d1, min(max(cur_d2-1,1),nd2)),          #(i,j-1)

          c(min(max(cur_d1+1,1),nd1), min(max(cur_d2-1,1),nd2)), #(i+1,j-1)

          c(min(max(cur_d1-1,1),nd1), min(max(cur_d2+1,1),nd2))) #(i-1, j+1)

  next_assig      <- next_assig[!duplicated(next_assig)]

  length_next_assig    <- length(next_assig)

  difference      <- c()

  for(ll in 1:length_next_assig)

  {

   difference  <- c(difference, abs(pijk[nd2-next_assig[[ll]][2]+1, next_assig[[ll]][1]]—target))

  }

  ind_min_dist <- which.min(difference)

  next_d1   <- next_assig[[ind_min_dist]][1]

  next_d2   <- next_assig[[ind_min_dist]][2]

  return(list(next_d1,next_d2))

 }

 ## nd1 et nd2 are numeric

 if(is.numeric(nd1)==FALSE) stop(“nd1 must be numeric”)

 if(is.numeric(nd2)==FALSE) stop(“nd2 must be numeric”)

 ## nd1 nd2 entier>0

 if(ceiling(nd1)-nd1!=0 | nd1<=0) stop(“nd1 must be Integer (>0)”)

 if(ceiling(nd2)-nd2!=0 | nd2<=0) stop(“nd2 must be Integer (>0)”)

 ## nd1 >= nd2

 if(nd1 < nd2) stop(“nd1 must be superior to nd2”)

 # For tox_true

 ## tox_true is matrix

 if(is.matrix(tox_true)==FALSE)stop(“fu must be a matrix (Numeric Argument)”)

 ## Proba of tox between 0 and 1

 if(min(tox_true)<0 | max(tox_true)>1) stop(“Verify probability tox_true (<0 or >1)”)

 rst_start_up <- start_up(start_up_end,ntot,ncoh_st,cur_d1,cur_d2,npts,true_tox,ntox)

 npts    <- rst_start_up$npts

 ntox    <- rst_start_up$ntox

 cur_d1  <- rst_start_up$cur_d1

 cur_d2  <- rst_start_up$cur_d2

 ntot    <- rst_start_up$ntot

 n_rem  <- rst_start_up$n_rem

 nused   <- rst_start_up$nused

 y    <- rst_start_up$y

 pijk    <- rst_start_up$pijk

 ### Two-Dimensional Trial

 for(i in 1:(n_rem %/% ncoh))

 {

  npts[nd2-cur_d2+1, cur_d1] <- npts[nd2-cur_d2+1, cur_d1] + ncoh

  true_tox          <- tox_true[(nd2-cur_d2+1),cur_d1]

  y_tox           <- gene_tox(ncoh, true_tox)

  y             <- c(y, sum(y_tox))

  ntox[nd2-cur_d2+1, cur_d1] <- ntox[nd2-cur_d2+1, cur_d1]+sum(y_tox)

  pijk            <- Estimation_pijk(nsamp, nd1, nd2, prior_1, prior_2, npts, ntox,Interaction)

  next_RD            <- Recommended_dose(cur_d1,cur_d2,pijk,target,npts,ntot,nd2)

  cur_d1            <- next_RD[[1]]

  cur_d2            <- next_RD[[2]]

 }

 ## target between 0 and 1

 if(min(target)<0 | max(target)>1) stop(“Verify probability of tox (<0 or >1)”)

 ### MTD

 difference <- abs(pijk-target)

 MTD_comb <- which(difference==min(difference,na.rm = T), arr.ind = T)

 MTD   <- c(MTD_comb[2],(nd2-MTD_comb[1]+1))#c(agentA,agentB)

 ### MTD by level

 MTD_lv  <- apply(difference,1,which.min)

 return(list(npts = npts, ntox = ntox, MTD = MTD,pijk = pijk,MTD_lv = MTD_lv))

}

ntot_used = 0

for(i in 1:nsim)

{

result = Design_Wang_Ivanova(nd1,nd2,tox_true,prior_1,prior_2,target,ntot,ncoh_st,ncoh,nsamp,Interaction)

result_MTD[(nd2-result$MTD[2]+1),result$MTD[1]]  <- (result_MTD[(nd2-result$MTD[2]+1),result$MTD[1]]+1)

result_pt                      <- (result$npts+result_pt)

result_tox                      <- (result$ntox+result_tox)

MTD_lv                       <- as.numeric(result$MTD_lv)

MTD_lv                       <- ifelse(is.na(MTD_lv),0,MTD_lv)

ntot_used                      <- ntot_used+sum(result$npts)

ntot_mean                      <- ntot_used/i

 for(ii in 1:nd2)

 {

  if(MTD_lv[ii]!=0)

  {

result_MTD_lv[ii,MTD_lv[ii]] <- (result_MTD_lv[ii,MTD_lv[ii]]+1)

  }

  }

}

Rst_MTD     <- (result_MTD*100)/nsim

Rst_MTD_lv   <- (result_MTD_lv*100)/nsim

Rst_pt      <- ((result_pt/nsim)/ntot_mean)*100

Rst_tox      <- ((result_tox/nsim)/ntot_mean)*100

# Imput Parameters

print(“+--------------------------+”)

print(“| Imput Parameters  |”)

print(“+--------------------------+”)

print(paste(“ - Number of dose level agent A (nd1):”,nd1))

print(paste(“ - Number of dose level agent B (nd2):”,nd2))

print(paste(“ - Prior distribution agent A (prior_1):”,paste(c(prior_1),collapse=“ ”)))

print(paste(“ - Prior distribution agent B (prior_2):”,paste(c(prior_2),collapse=“ ”)))

print(paste(“ - Target DLT Rate (target):”,target))

print(paste(“ - Nb of patients enrolled (ntot):”,ntot))

print(paste(“ - Nb of patients enrolled in the cohort used for the starting rule (ncoh_st):”,ncoh_st))

print(paste(“ - Nb of patients enrolled in the cohort used for the main design (ncoh):”,ncoh))

print(paste(“ - Number of iteration needed for the MCMC (nsamp):”,nsamp))

print(paste(“ - Number of simulation (nsim):”,nsim))

print(“+--------------------------+”)

print(“|  Output Parameters |”)

print(“+--------------------------+”)

print(“ - Average number of patients treated at the test doses in terms of percentage (Rst_pt):”)

print(round(Rst_pt,3))

print(“ - Average number of toxicities seen at the test doses in terms of percentage(Rst_tox):”)

print(round(Rst_tox,3))

print(“ - Distribution of the MTD estimates in terms of percentage(Rst_MTD):”)

print(round(Rst_MTD,3))

print(“ - Distribution of the MTD estimates for each dose level of the agent B in terms of percentage (Rst_MTD_lv):”)

print(round(Rst_MTD_lv,3))

print(“+--------------------------------------------------------------------+”)

print(“| Send feed back via e-mail:              |”)

print(“|  —RAJOUTER TON MAIL          |”)

print(“+--------------------------------------------------------------------+”)

return(list(Rst_MTD = Rst_MTD,Rst_pt = Rst_pt,Rst_tox = Rst_tox,Rst_MTD_lv = Rst_MTD_lv,ntot_mean = ntot_mean))

}

**Example**

For example, for scenario 1, the parameters should be be fixed in the R function as:

tox_true ← matrix(c(0.08, 0.13, 0.20, 0.29, 0.40, 0.53, 0.05, 0.08, 0.13, 0.20, 0.29, 0.40, 0.03, 0.05, 0.08, 0.13, 0.20, 0.29), byrow = T, nrow = 3)

prior_1 ← c(0.05, 0.1, 0.2, 0.3, 0.5, 0.7) # 6 dose levels of agent A

prior_2 ← c(0.05, 0.1, 0.2) #3 dose levels of agent B

target ← 0.2

ntot ← 54

ncoh_st ← 2

ncoh ← 3

nsamp ← 1

nd1 ← 6

nd2 ← 3

seed ← 2

nsim ← 4000

Interaction ← 0

# Generate 4000 replicates of the two-dimensional dose-finding design of Wang and Ivanova with 54 subjects

result_sc = SIM_design(nd1, nd2, tox true, prior 1, prior 2, target, ntot, ncoh st, ncoh, nsamp, nsim, seed)

### Application: Validity of R scripts for Wang and Ivanova approach

To validate our R scripts, we ran the same scenarios (dose-toxicity relationships) proposed by Wang for the simulation study. The results were compared to those published in their paper. The results using R function were similar to those published by Wang.

Using the R functions, the results on the percentage of DLTs observed, and a recommended MTD at the end of the trial can be also obtained. The MTD is defined as the dose combination associated with a probability of DLT closest to the target toxicity (results available on request).

## Conclusions and discussion

As stated by Hirakawa et al., thus far, there are no silver bullet designs to resolve the two-agent dose-finding problem. The operating characteristics of model-based dose-finding methods may be varied depending on prior toxicity probabilities, prior distributions, and number of patients included in trials. It is recommended to assess the performance of the designs in the context of the clinical trial before beginning the trial. The two-dimensional dose-finding design proposed by Wang and Ivanova is a comprehensive approach that yields good performances. The two R functions that we propose can facilitate the use of this design in practice. With the first function, the design can be applied to an actual phase I trial. With the second function, a simulation study can be run in order to calibrate the design before its application for a phase I trial, or in order to compare it with other designs. Both R functions are simple, and each part of the algorithm (start-up, decision rule…) can be modified depending on the requirements of the trial: i) the start-rule can be updated depending on the clinical context, ii) even if the design proposes to identify a set of MTDs, one MTD can be chosen from the set. For example, the MTD with a probability of DLT closest to the target toxicity can be chosen, or clinicians may prefer to choose to identify the dose for further trials depending on the clinical context.
